# Supplementary figures and images for: Dynamic coefficient symmetric polynomial-based secure key management scheme for Internet of Things (IoT) networks
Source: PeerJ Comput Sci. 2023 Dec 15;9:e1726. doi: 10.7717/peerj-cs.1726 (PMC10773850; doi:10.7717/peerj-cs.1726)

Remote Server Nodes

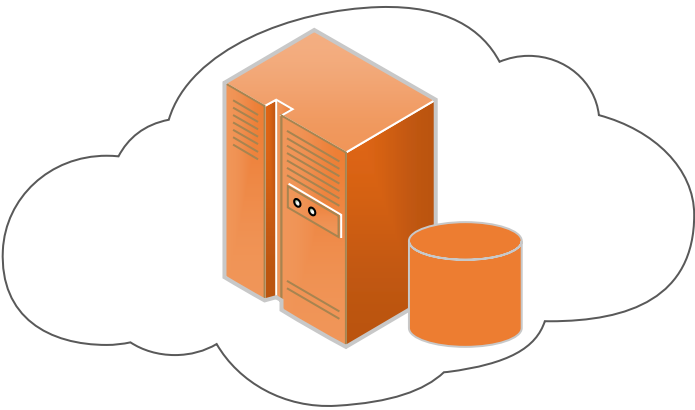

Gateway Nodes

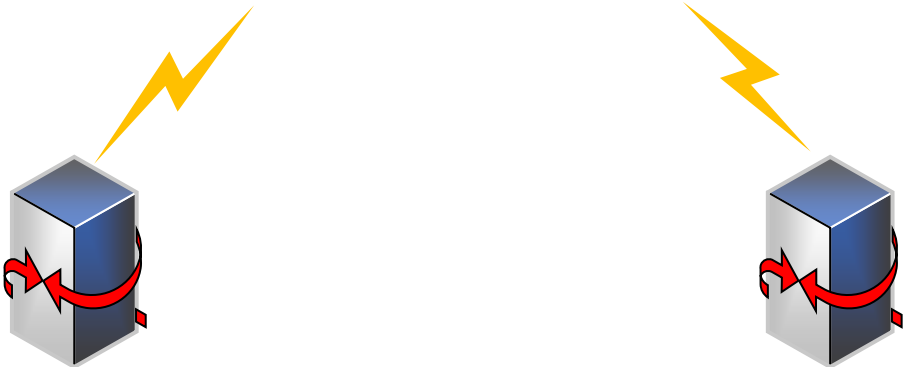

Constrained Nodes

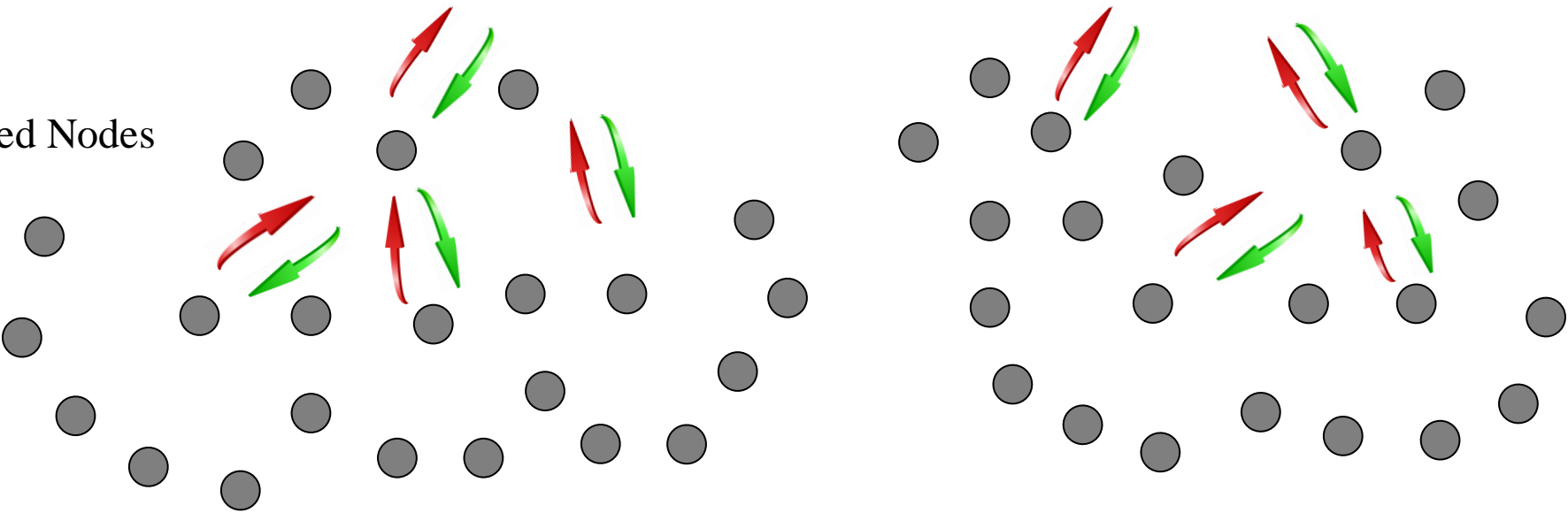

Supplement: Supplemental Information 1 [file peerj-cs-09-1726-s001.zip › Raw data andor code/1/1.pdf]

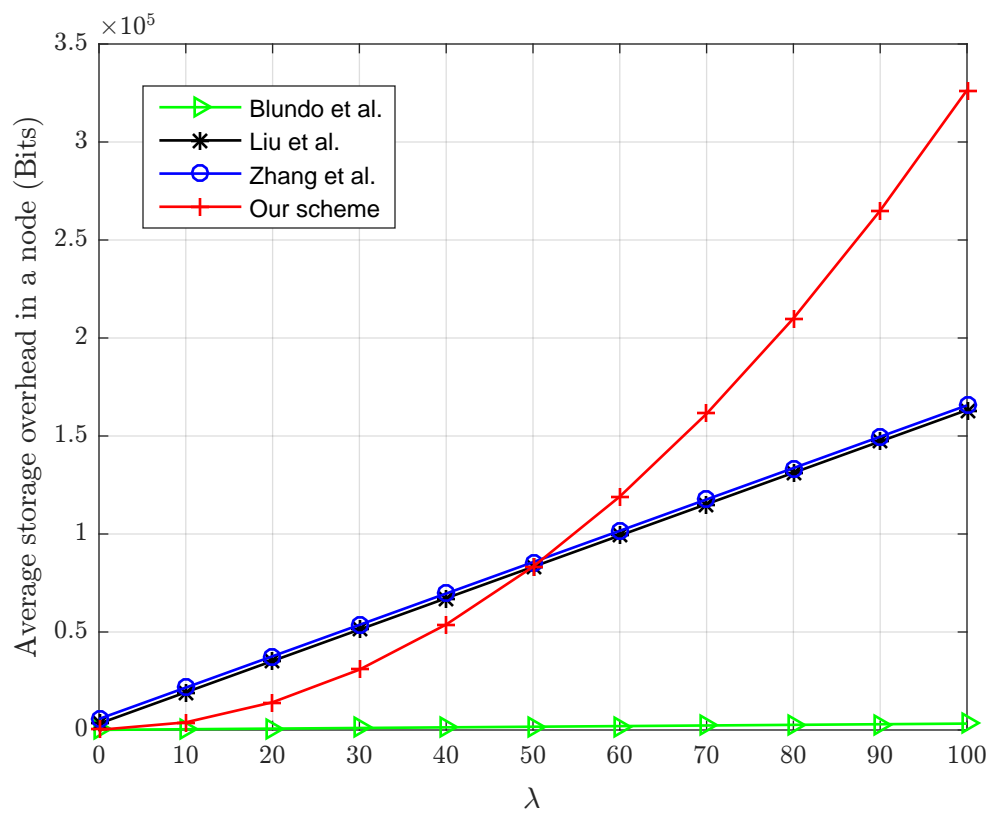

Supplement: Supplemental Information 1 [file peerj-cs-09-1726-s001.zip › Raw data andor code/10/10.pdf]

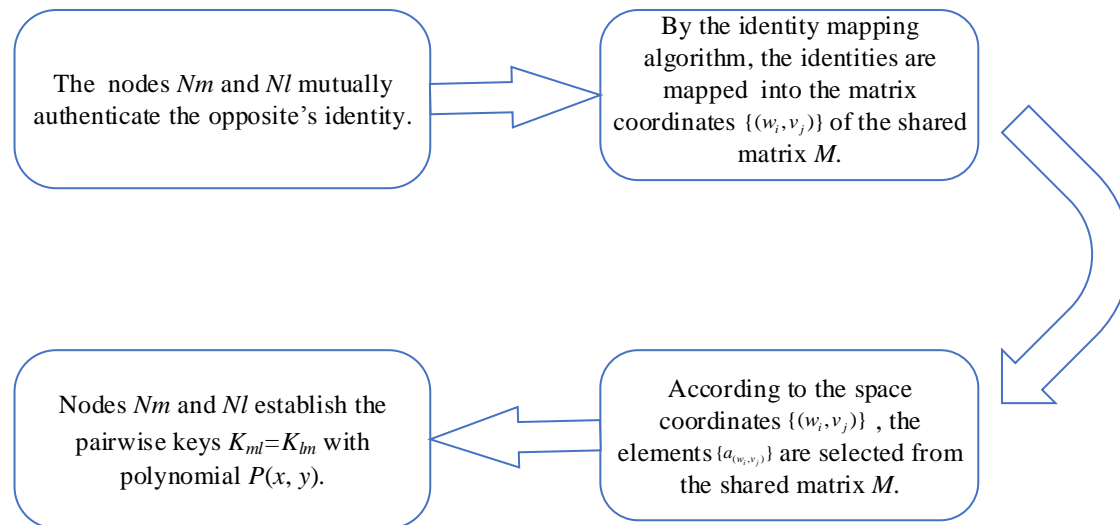

Supplement: Supplemental Information 1 [file peerj-cs-09-1726-s001.zip › Raw data andor code/2/2.pdf]

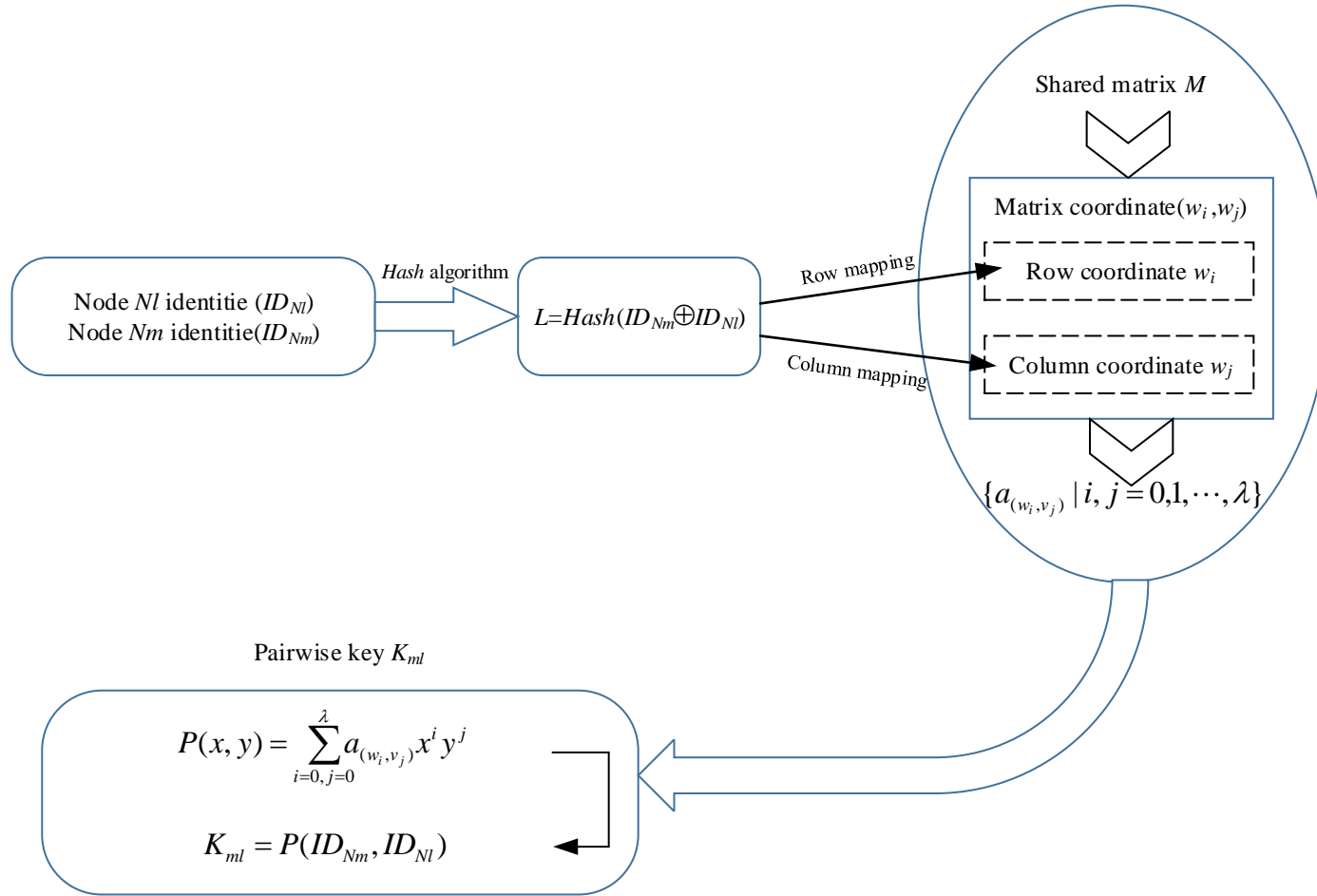

Supplement: Supplemental Information 1 [file peerj-cs-09-1726-s001.zip › Raw data andor code/3/3.pdf]

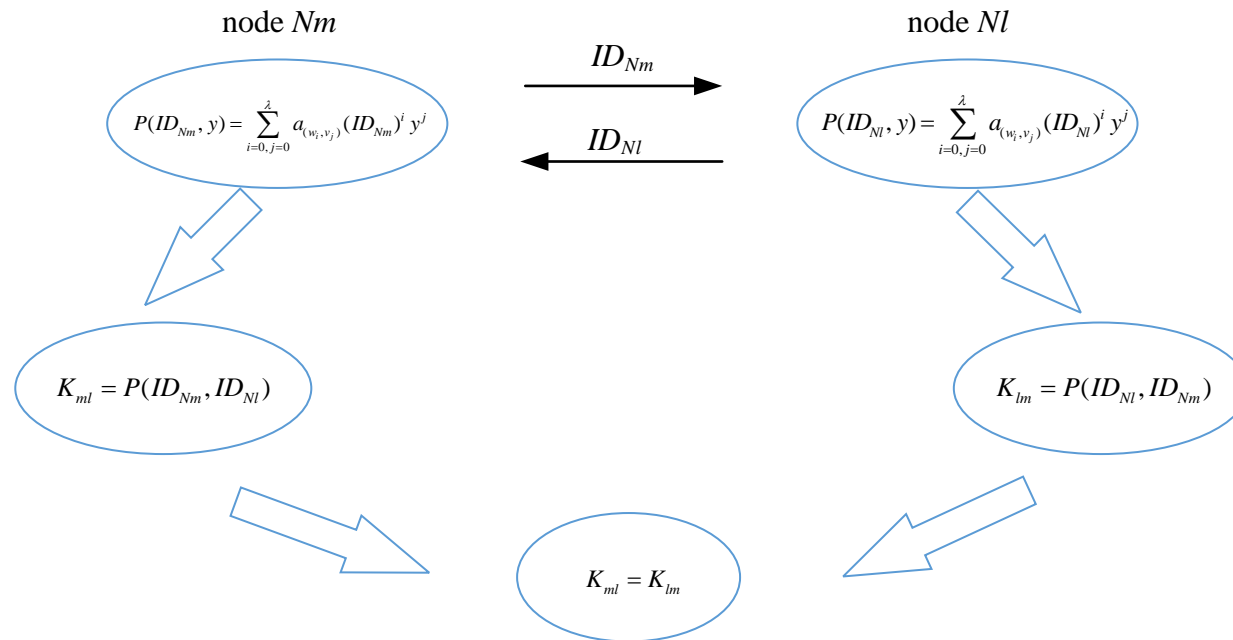

Supplement: Supplemental Information 1 [file peerj-cs-09-1726-s001.zip › Raw data andor code/4/4.pdf]

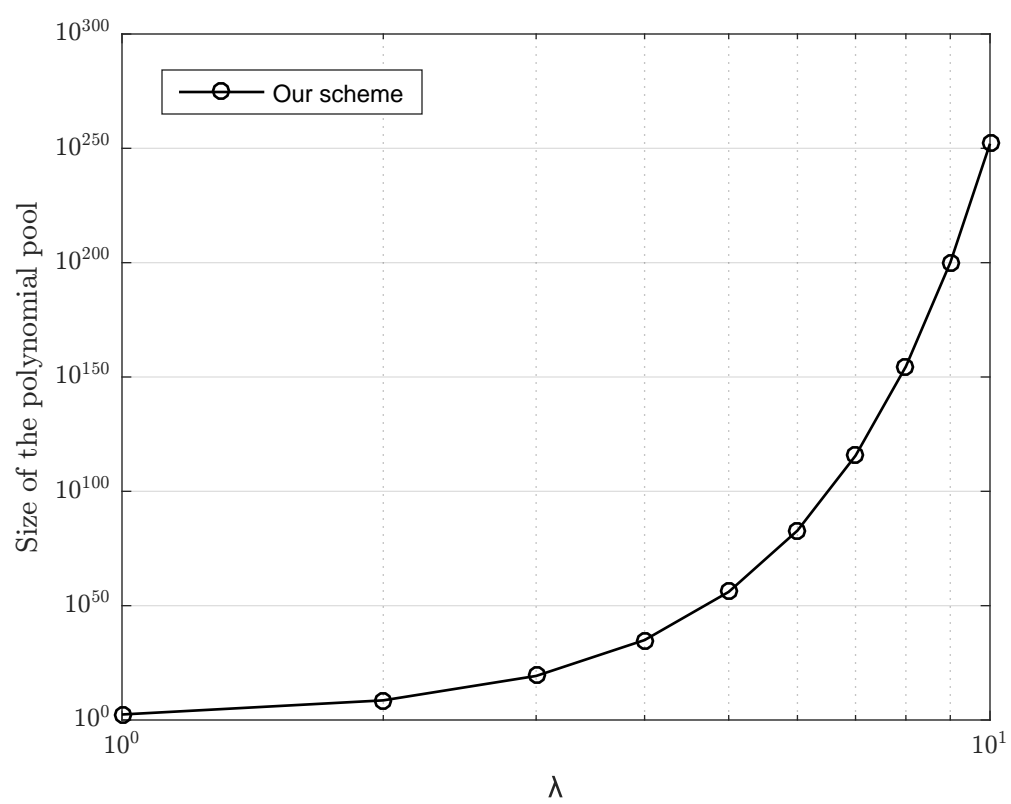

Supplement: Supplemental Information 1 [file peerj-cs-09-1726-s001.zip › Raw data andor code/5/5.pdf]

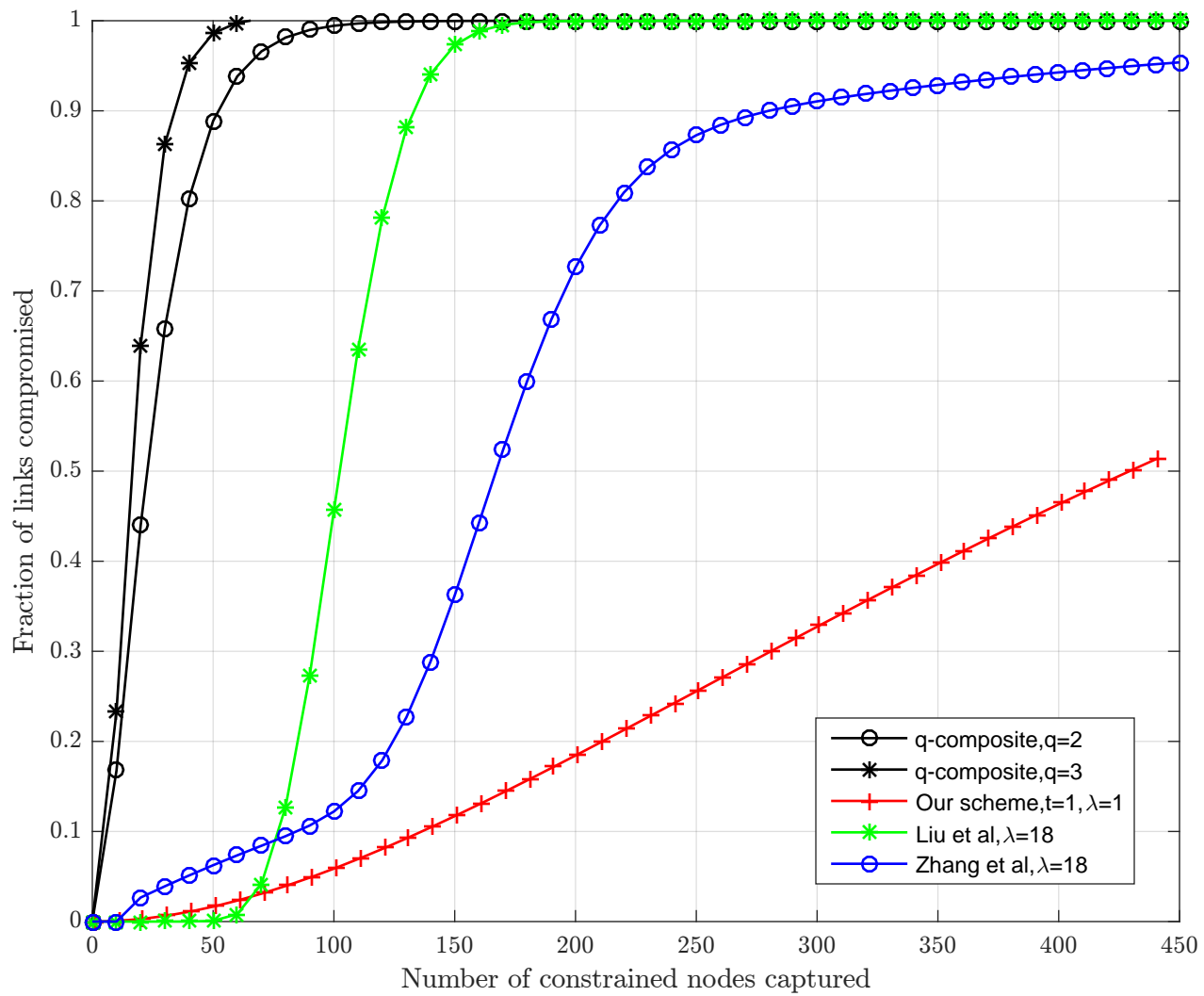

Supplement: Supplemental Information 1 [file peerj-cs-09-1726-s001.zip › Raw data andor code/6/6.pdf]

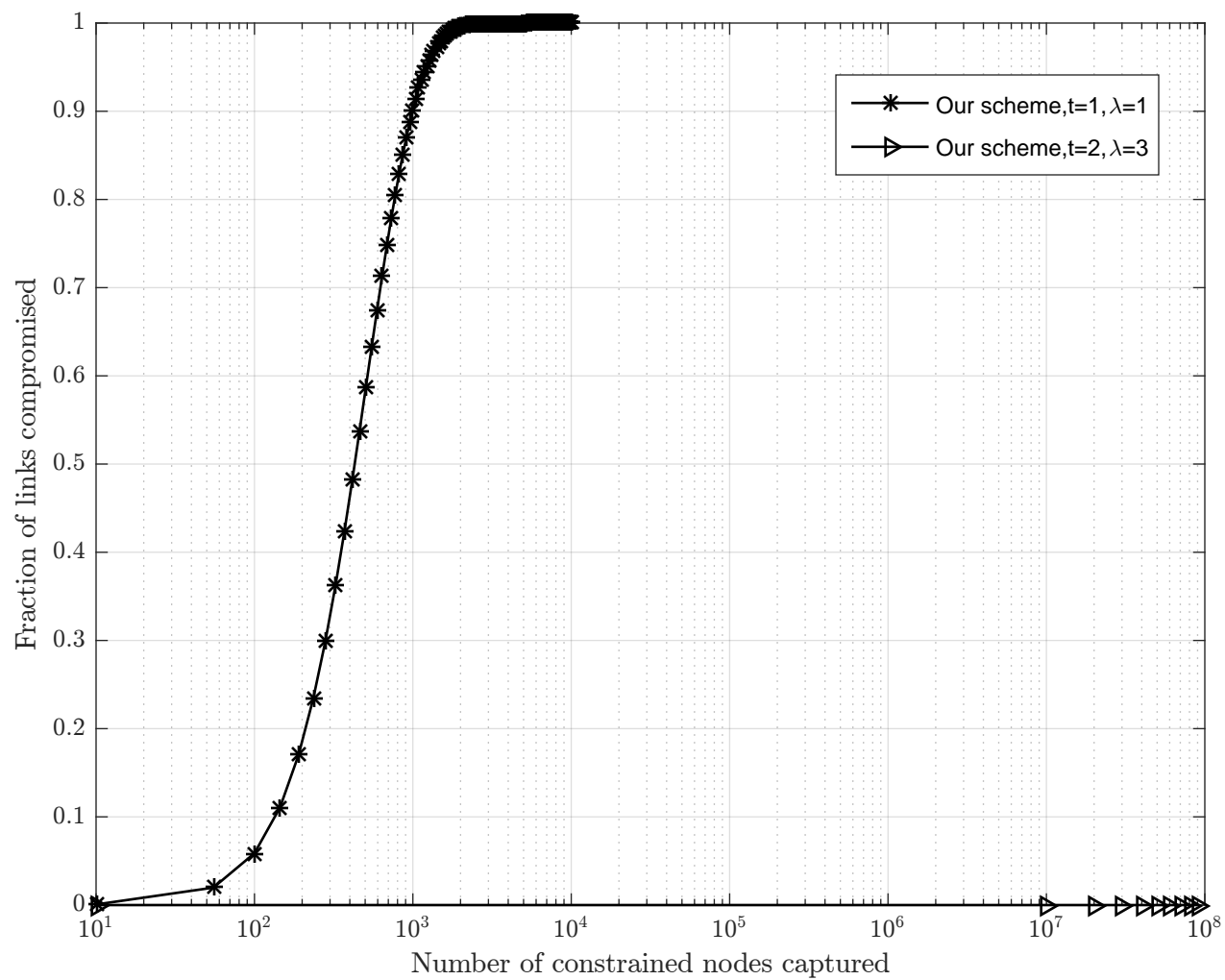

Supplement: Supplemental Information 1 [file peerj-cs-09-1726-s001.zip › Raw data andor code/7/7.pdf]

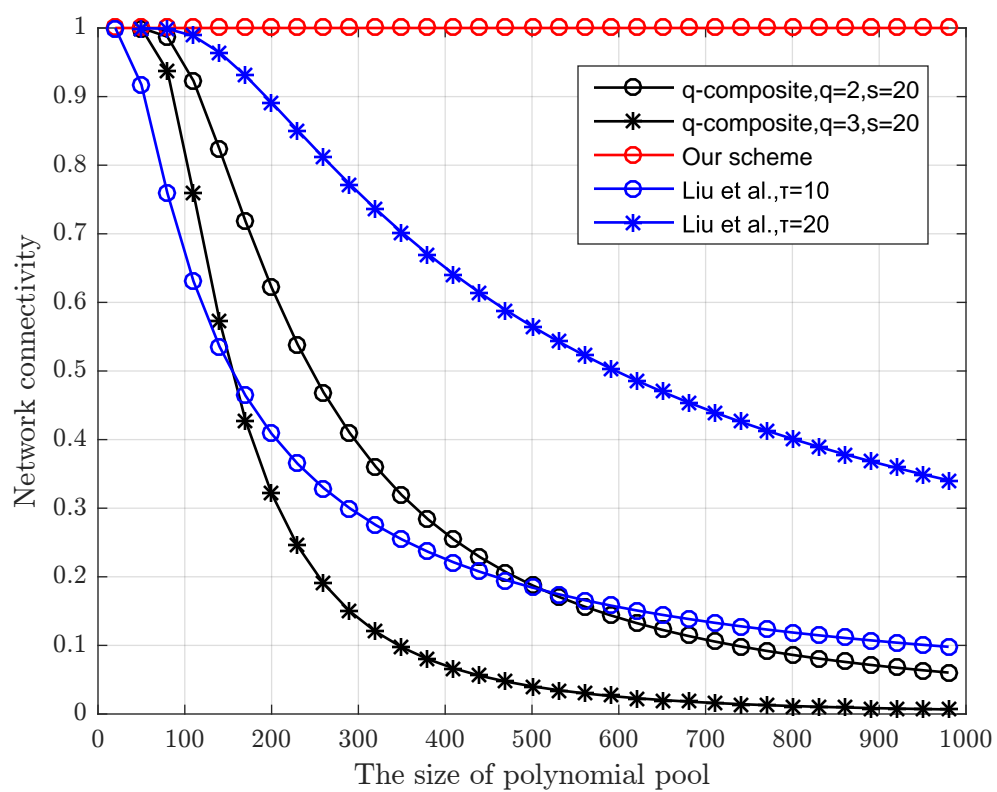

Supplement: Supplemental Information 1 [file peerj-cs-09-1726-s001.zip › Raw data andor code/8/8.pdf]

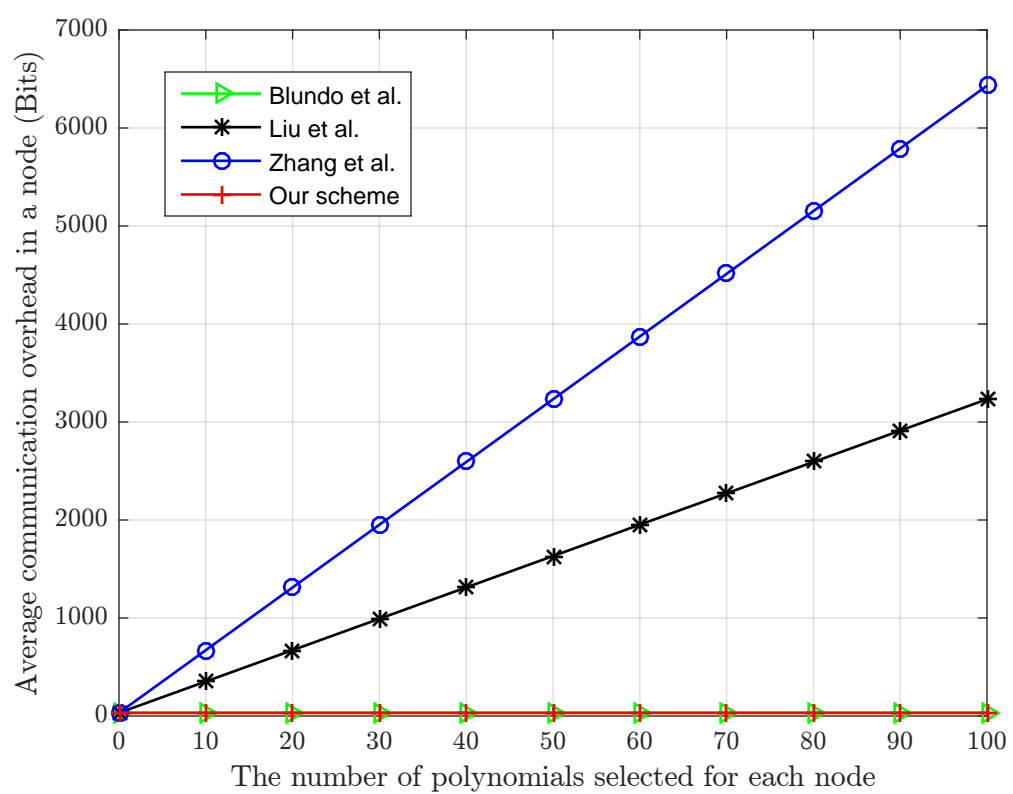

Supplement: Supplemental Information 1 [file peerj-cs-09-1726-s001.zip › Raw data andor code/9/9.pdf]
